# Southern Branch, National Dental Association, Second Annual Meeting, New Orleans, La., February 9, 10, 11 and 13, 1899

**Published:** 1899-06

**Authors:** 


### PROCEEDINGS.

### Southern Branch, National Dental Association, Second Annual Meeting, New Orleans, La., February 9, 10, 11 and 13, 1899.

[*Continued from May No., Page* 236.]

### MICROSCOPY, HISTOLOGY AND BACTERIOLOGY.

The chairman of the committee, Dr. W. H. Martin, Yazoo City, Miss., presented a brief report on the work of the year in these branches, which was discussed by Drs. George B. Clement, W. C. Barrett, J. Y. Crawford, L. M. Cowardin, Laurence P. Leonard, Waseca, Minn.; Jas. S. Knapp and Wm. Ernest Walker.

DISCUSSION.

DR. CLEMENT said that unfortunately the great body of dental practitioners are hampered by lack of equipment with which to carry on such investigations; they can only accept the teachings of such men as Miller, Black, Williams and others. While it is true that all teeth are alike subject to infection, it is also true that all teeth do not suffer alike from the ravages of decay. All are alike susceptible to decay, provided the micro-organisms are permitted to find lodgment, provided with nutriment, and allowed to remain undisturbed. This being the status, our duty in the premises is clear: they must not be allowed to remain undisturbed; they must not be provided with nutriment; they must be destroyed. How is this to be effected? By cleanliness, by sterilization—by oral hygiene.

DR. W. C. BARRETT gave a very clear and elaborate exposition of Dr. Miller's theory of dental caries, the germ theory, and of the later investigations of Black and Williams. He said: Miller made the discovery which solved the problem of the centuries.

All the later investigators are working on the foundation laid by Miller. Valuable as is their work, they could have done nothing except upon the basis of Miller's discovery. And yet I think they all make the common mistake of excluding the vital principle as a factor in pathological degeneration.

Dr. J. Y. Crawford said that with all due respect to Dr. Miller, he did not believe that caries produced in the laboratory, in teeth out of the mouth, would be identical with the process taking place in the mouth in living tissues. Vitality must play an important part in the etiology of dental caries. Twenty years ago it was called a chemico-vital process, and it is that yet. He said, or it has been said here, that some teeth are harder than others, some teeth softer than others. I go further than that. I say there is hardly a tooth in the world, a living tooth, a tooth with a living pulp, that is not harder at some times than at others; that may not be different to-day from what we found it last year. These changes occur only in a tooth with a living pulp. Why does decay occur bilaterally?

Dr. Barrett: Bacteriology shows the reason.

Dr. Crawford: I want to get hold of that kind of bacteriology! There may be something valuable in it, but great minds the world over are capable of making mistakes.

Dr. Cowardin: I attribute to vitality all the emphasis that Dr. Crawford gives it, but there are times when vital force fails to perform its functions in the most perfect manner. This is especially true in regard to the teeth. In dental caries vital force fails to perform its function in the perfect formation of secretions which should prevent the presence of the fungi which causes caries. Therefore we must keep on fighting with the germicides, antiseptics, etc.

Dr. L. P. Leonard: Whoever has followed up the investigations of Miller, Williams and Black, these men who work through the long hours of the night for our benefit, feel that they will find their reward in the manifested appreciation of those who have comprehended their work and who can appreciate what the microscope reveals. Whether teeth are hard or soft, they can be sound if the patient will second our efforts with the brush, floss and antiseptics. With good fillings and proper hygienic care the worst teeth may be saved.

DR. JAS. S. KNAPP: The decay-producing bacteria may be present in the mouth, but they can not get in their work unless conditions invite them. There must be some sheltered, defective spot where they may lodge undisturbed and which retains its food—debris upon which they are nourished. They cannot attack sound, clean enamel.

DR. WM. ERNEST WALKER: The difference between the various aspects of this question is more apparent than real. The trouble is each one has in view a different stage or phase of the process. One is right in saying that imperfect development constitutes an important factor in decay of the teeth. Another is right in attributing this imperfect development to an arrest of or imperfect nutrition at some period in the formation of the teeth, the defect being naturally bilateral, and hence decay is bilateral. But all this argues nothing against the agency of bacteria in causing the decay. It is also true that the bacteria, though present in the mouth, do not attack sound, smooth, clean enamel. I doubt that it is practicable to keep the mouth absolutely sterile by the use of chemic antiseptics and germicides. Friction, gently but persistently applied with a stiff brush, will do more to prevent dental caries than mouth-washes.

DR. JAS. S. KNAPP: The great original cause is what I have termed "imperfect dentification." The teeth are imperfectly organized, imperfect in structural qualities.

DR. J. Y. CRAWFORD: We can secure immunity from decay, but it must be by local applications and local treatment. We must destroy the organisms which produce the toxins which create the disease.

### A REMARKABLE CASE.

BY. C. B. JOHNSTON, MONROE, LA.

Abstract of paper read.

During the Civil War, at the siege of Vicksburg, on the 10th of June, 1863, Capt. Alex. Myatt, of the Confederate forces, received what was supposed to be a fatal wound, having been shot in the right eye, the missile afterward proving to be a Springfield rifle-ball of about 55 caliber. Death did not ensue, but for more than a third of a century the leaden missile was a continual source of torture to its victim. The wounded soldier remained in the hospital until the fall of Vicksburg on the 4th of July. General Grant, who was in command of the Federal forces, visited the Confederate hospital after the surrender, and had his attention called to the nature of the wound, and became so interested in the case that he ordered his staff surgeon to visit the patient and examine the wound. After careful examination, this eminent surgeon agreed fully with the diagnosis made by the Confederate surgeons: that the bullet, after penetrating the eye, had taken a backward course, lodging in the back part of the head, the location making its removal impossible except at the risk of the patient's life. On the 17th of July the patient was paroled and sent home, a physical and also a financial wreck. Notwithstanding his condition, however, he has accumulated a competency for his declining years and made ample provision for his family.

During these thirty-five years he suffered more or less constantly from the presence of the bullet, and from time to time visited the most eminent surgeons in hope of obtaining relief, among others the noted Dr. Stone, of New Orleans, but always to no avail.

His sufferings were finally brought to an end by the services *of a dentist*, and in a most remarkable manner. In the fall of 1898 Capt. Myatt called on Dr. Johnston for the removal of a broken tooth. To the surprise of the operator a foreign substance, evidently *lead*, was found to be occupying the depths of the socket after the removal of the tooth. This seemed so incredible that, at the request of the patient, his family physician was called in to make an examination. A brother dental surgeon, Dr. J. F. Johnston, was also called in. After careful examination the foreign substance was pronounced to be the bullet which had entered the eye of the patient thirty-five years previous.

The operation of removal was tedious and difficult, as it was necessary with saws and drills to divide the bullet into sections, and several days were consumed in the operation, as the patient objected to the use of any anaesthetic. It was successfully accomplished, however, and the old veteran enjoys life once more and has gained twenty pounds in weight since the operation.

After the removal of the several pieces into which the bullet was divided, the gaping wound was well washed out with peroxid of hydrogen and then packed with dry cotton thoroughly impregnated with iodoform, this dressing being changed once a week. Some little necrosed bone was thrown off, and there is still a little more; but as the patient will not permit the use of any anaesthetic, Dr. Johnston proposes simply to assist nature in completing the process of healing. One small piece of necrosed bone came away through the nostril. The patient is doing well and has better health and enjoys life more than at any time since receiving the wound in June, 1863.

### "ABSORPTION AREAS" AND "PULP NODULES."

BY SAMUEL P. COWARDIN, D.D.S.

Abstract of paper read at the New Orleans meeting.

In making a very large number of sections, in his microscopic work, not less than three thousand human teeth having been carefully inspected, three teeth have been found by the author containing large absorption areas. In one of these teeth (which was submitted for inspection) there is on the outer surface a seeming plug of cement-like substance which seems to fill the point from which the tooth was attacked, the partly refilled absorption area connecting with the cementum and not with the pulp. In these three cases some considerable part of the dentin is absorbed, but in quite a good many cases, probably fifty or sixty, the author has found minute "absorption areas," and he has observed that whenever he cuts an old tooth which shows a heavy coat of cementum with evidence of abscess, there is more or less of absorption area. The author suggests the desirability of an investigation as to what proportion of normal teeth contain pulp stones. Many of the teeth used by the author have probably been extracted for purposes of regulating or inserting artificial dentures, and in these apparently normal teeth he finds pulp stones very common, probably 66 percent showing pulp stones. The author's observations lead him to conclude that in fully developed teeth, and certainly in old teeth, it is rare that a pulp is entirely free from these nodules. Hence he doubts if they are so generally a source of the serious trouble attributed to them by many writers.

### FORMALDEHYDE IN DENTISTRY.

BY H. STUART M'LEAN, M.D., RICHMOND, VA.

Abstract of paper read.

This gives the results of the first of a series of systematic tests undertaken for the purpose of determining definitely what has hitherto been surmised, or taken for granted, as to the action of certain germicidal agents and their exact value as sterilizing agents for dental instruments, tooth cavities and root canals.

The role of bacteria in the production of various diseases of the teeth is indisputable, and in dental pathology the classic researches of Miller rank with the studies of the greatest medical pathologists. The part played in the causation of caries, either by acid formation or by some other product, and the production of pulpitis, either by direct exposure or by bacterial infection through the dentinal tubules, is fully established, and there remains simply the work of studying the various agents which may be used to counteract these conditions.

The first of the present series of test experiments was made with formaldehyde, which has been recommended for use in dentistry, but accompanied only by general observations as to its action. We are informed, for instance, that such and such an antiseptic "inhibits the growth bacteria when used in proper strength," but this simply means that when the restraining agent is removed the growth continues as before. Again, to say that the germs are "destroyed" by a certain agent, and omit *the time*

required to consummate this end, is to omit a very essential detail. In the present series of tests, upon instruments, teeth *in situ* and extracted teeth in the bacteriological laboratory, the *time* required for the given action is made a special point of observation.

(1) STERILIZATION OF INSTRUMENTS.

In the tests with instruments, burs, pluggers, excavators, etc., were used, and into the crevices and serrations cultures of staphylococcus pyogenes aureus and bacillus typhosus were plastered, after which the inoculated portions were rolled in a paste of "tooth-dust" and saliva (*a*). They were then placed in the incubator and dried, thus imitating as closely as possible the condition of an instrument after use and before cleansing. Others (*b*) were dipped in a thirty-six-hour bouillon culture of the same bacteria and dried in a similar manner. The instruments were next immersed in a 5 percent solution of formalin, and after immersion of from five to thirty minutes were, where size permitted, placed in a tube of bouillon, or cultures taken by scraping the instruments, or stroke cultures made in agar-agar. Those prepared by the first method (*a*) were rinsed in sterile water after removal from the formalin solution; the others (*b*) were pressed lightly for a moment on sterilized filter paper. Control experiments were made with all the tests. With immersion in formalin, 5 percent, for five and ten minutes, results varied slightly, but thirty minutes immersion gave the result "sterile" in every case. In five minutes, of five cultures of staph. pyog. aureus two showed scanty growth, one in sixty, the other in seventy-two hours; while of ten minutes, one of four cultures showed scant growth in five days; twenty minutes, "sterile." The variation was probably due to the hardened condition of the material with which the instruments were plastered, as no agitation was used while exposing the instruments to the action of the antiseptic. With twenty minutes immersion, in one tube only (of eight) bacillus typhosus showed growth on the third day; but, as this grew as well and as rapidly as the control cultures, accidental contamination is strongly suggested. One objectionable feature which developed was the tendency to rust, of instruments left in the solution over night, eighteen hours or more; but as only old instruments, roughened by previous usage, were used in these experiments, oxidation was probably favored by these conditions. The practical inferences from these experiments was, (1) that all debris should be removed from instruments before they are laid aside for subsequent sterilizing, as the caked debris may harden and prevent the penetration of antiseptic fluids. (2) The instruments should be dried after cleansing and not allowed to remain longer than necessary in the solution. (3) A fresh solution is advisable every morning unless precautions are taken to prevent evaporation. Formaldehyde is an inexpensive sterilizing agent. (4) The solution is most convenient for use if kept in a shallow glass dish with flat bottom and perpendicular sides, with a loosely-fitting cover.

(2) STERILIZATION OF CAVITIES.

For cavities in teeth, *in situ*, molars were selected and carefully isolated from the cheek and tongue. The cavity was carefully cleared of all extraneous matter, decay, etc., and prepared as for filling. After thoroughly drying with cotton, with a platinum needle a small portion of food removed from the cavity, and which had been soaked for a few minutes in a thirty-six-hour bouillon culture of the staphylococcus pyogenes aureus (or of bacillus typhosus), was smeared on the bottom and sides of the cavity. The cavity was immediately filled with cotton saturated in a 5 percent solution of formalin, which was left in position for five minutes. The cotton was then removed and cultures taken from the bottom and sides of the cavity. Cotton with a 1 percent solution was then introduced and the cavity sealed, some for twelve and some for twenty-four hours, when cultures were again taken from the surfaces.

The technique with extracted teeth was practically the same. The tabulated results show that sterilization takes place in the majority of instances after a five minutes exposure; often twelve and twenty-four hours with 1 percent results uniformly successful.

(3) STERILIZATION OF ROOT CANALS.

The time allotted to the root canal tests was too brief for any accurate conclusions to be reached, the chief obstacle being the difficulty in obtaining from the bottom of the canal a culture that would not be removed from the fine instrument by friction against the sterilized material at the top of the canal. The method employed was practically the same as for cavities. While marked success was obtained in one series of tests, uniform failure attended another series, under apparently the same conditions. As this is, in many respects, the most important of the three divisions of the subject, on account of the danger of forcing infectious material through the apical foramen, thereby producing pericementitis, abscesses, and even distant metartatic septic lesions, a more thorough investigation has been planned, with a radical change in the technique, and with various antiseptics, in the hope of arriving at some reliable conclusion.

DISCUSSION.

In the discussion of this paper, Dr. Walker spoke of the desirability of a definite basis from which to reach conclusions as to the positive merits of these agents, formalin being the one in question at this time; how long it takes to penetrate to the foramen, sterilizing the canal throughout, etc. If we knew that in ten, twenty or thirty minutes a superior root canal be completely sterilized, with no liability of infecting the antrum, that knowledge would be very satisfactory to us. We know that merely wiping out the canal is no safeguard. If we knew that our instruments could be effectually sterilized by immersion in some solution, that would be much less trouble than boiling them. But we want these points proved, not merely asserted. Hence the value of such experiments as those described in the paper, which are but the beginning of a series to be continued until definite and positive results are reached or found to be impossible.

Dr. H. H. Johnson considers formaldehyde as too powerful an irritant to be used in a pulp chamber or a root canal, from the liability of penetrating the foramen and causing serious trouble.

Dr. MacLean considers that there is very little danger of its escaping through the apical foramen, its coagulating properties preventing that. But in all the experiments upon teeth *in situ* they were very carefully isolated. He would, however, consider over 5 percent too strong for use in the teeth except under unusual conditions.

Dr. Johnson cited a case in his own personal experience. Having taken into his mouth from a sample bottle of mouth-wash, and finding it very hot, he looked at the formula and found that it contained 5 percent formaldehyde.

Dr. Walker disclaimed against the fairness of citing this this incident against the use of formaldehyde in cavities in the teeth and in root canals which are properly isolated from the soft tissues. He has been using formaldehyde empirically for the last two years, having, however, first read all the accessible literature on the subject in order to use it as intelligently as possible. He has had no deleterious effects, but apparently good results. In the ordinary dressing of root canals he uses 3 percent, but in cases of open foramen only 2 percent. When there is reason to believe the foramen small, and prompt results are desired, he uses 6 percent, keeping solutions of different strengths on hand, ready for use as required. Ten percent may be applied to the putrescent contents of a root canal, the strength being reduced as it penetrates the mass of putrescent matter.

